# Prediction model for intrapartum labor analgesia efficacy based on preoperative multidimensional indicators

**DOI:** 10.3389/fmed.2026.1794753

**Published:** 2026-05-19

**Authors:** Biyao Wang, Wensheng He

**Affiliations:** 1 Wannan Medical College, Wuhu, China; 2 Department of Anesthesiology, The Second People’s Hospital of Hefei, Hefei, China

**Keywords:** epidural analgesia, labor analgesia, machine learning, prediction model, preoperative indicators

## Abstract

**Background:**

Effective labor analgesia is paramount for maternal well-being and positive birth experiences. However, the efficacy of intrapartum labor analgesia exhibits considerable inter-individual variability. Identifying reliable preoperative predictors for labor analgesia efficacy is crucial for optimizing pain management strategies and enhancing patient outcomes. This study aimed to develop and validate a prediction model for intrapartum labor analgesia efficacy utilizing preoperative multidimensional indicators.

**Methods:**

This retrospective study enrolled 137 parturients who received labor analgesia. Preoperative multidimensional indicators, encompassing demographic data, clinical characteristics, and psychological assessments, were systematically collected. Logistic Regression (LR), Naive Bayes (NB), Random Forest (RF), Support Vector Machine (SVM), and Extreme Gradient Boosting (XGB) models were developed and comparatively analyzed. Feature selection was conducted using LASSO regression. Model performance was rigorously evaluated using metrics such as Area Under the Curve (AUC), sensitivity, specificity, F1 score, positive predictive value, negative predictive value, calibration curves, and decision curve analysis.

**Results:**

Initial logistic regression analysis revealed several significant preoperative predictors, including pregnancy-induced hypertension (PIH), binary ultrasound cervical length, and continuous ultrasound cervical length. LASSO regression further refined the feature set, illustrating the dynamic changes in coefficients across varying lambda values. Among the developed models, the XGB model consistently demonstrated superior discriminative ability, achieving an AUC of 0.875 (95% CI: 0.802 to 0.948) in the training set and 0.759 (95% CI: 0.606 to 0.912) in the validation set, thereby indicating robust performance. Calibration curves provided insights into the agreement between predicted and observed probabilities across models. Decision curve analysis elucidated the clinical utility of these models, underscoring the potential net benefit of employing these predictive tools.

**Conclusion:**

This study successfully developed and validated a prediction model for intrapartum labor analgesia efficacy grounded in preoperative multidimensional indicators. The XGB model exhibited promising performance, suggesting its potential for clinical application in identifying parturients who could benefit from individualized pain management strategies. Further prospective validation in diverse cohorts is warranted to confirm these findings and facilitate clinical implementation.

## Introduction

Labor pain is recognized as one of the most intense forms of pain experienced by women, necessitating effective analgesia to improve maternal satisfaction and mitigate the physiological stress associated with childbirth (1). Neuraxial analgesia, particularly epidural analgesia, stands as the gold standard for pain relief during labor due to its superior efficacy (2). Despite its widespread effectiveness, the degree of pain relief can vary substantially among individuals, with some parturients experiencing inadequate analgesia or breakthrough pain (3). Inadequate labor analgesia may be associated with adverse clinical consequences, including disturbed labor progress, unfavorable maternal and neonatal outcomes, and negative maternal psychological outcomes, including an increased risk of postpartum depressive symptoms (4).

Prior research has investigated various factors influencing labor analgesia efficacy, including demographic attributes, obstetric history, psychological states, and genetic predispositions (5, 6). However, a comprehensive model that integrates multidimensional preoperative indicators to predict intrapartum labor analgesia efficacy remains an area requiring further exploration. Machine learning methodologies have demonstrated significant potential in constructing robust predictive models across diverse medical domains, owing to their capacity to unravel complex interactions among multiple variables (7, 8). Recently, several studies have successfully applied machine learning algorithms to predict various obstetric and anesthetic outcomes, such as intrapartum fever and neuraxial block efficacy, further highlighting their clinical utility in modern obstetrics (9).

This study endeavors to develop and validate a machine learning-based prediction model that leverages preoperative multidimensional indicators to forecast the efficacy of intrapartum labor analgesia. By pinpointing key predictors and rigorously evaluating the performance of various machine learning algorithms, we aim to furnish clinicians with a valuable instrument for optimizing pain management strategies and enhancing the overall labor experience for parturients.

## Methods

### Study design and participants

This retrospective cohort study included 137 parturients who received labor analgesia. The inclusion criteria were: (1) singleton term pregnancy (gestational age ≥ 37 weeks); (2) cephalic presentation; and (3) maternal request for and administration of epidural labor analgesia. The exclusion criteria included: (1) contraindications to neuraxial anesthesia; (2) severe maternal cardiovascular, hepatic, or renal dysfunction; and (3) missing critical baseline data exceeding 20%. This retrospective study was approved by the Ethics Committee of The Second People’s Hospital of Hefei (Approval No. [2025KY089]). The requirement for informed consent was waived by the Ethics Committee due to the retrospective nature of the study and the use of fully de-identified data.

### Labor analgesia procedure

At our institution, labor epidural analgesia is routinely administered by experienced anesthesiologists according to a standardized protocol. Epidural catheterization is typically performed using a conventional lumbar epidural technique. After confirmation of correct epidural placement, an initial loading dose of local anesthetic combined with opioid is administered. Analgesia is subsequently maintained using a standardized regimen, with adjustment according to maternal pain score, sensory block level, and labor progress. Rescue boluses are provided when breakthrough pain or inadequate analgesia occurs.

### Data collection

Preoperative multidimensional indicators were extracted from electronic medical records. These indicators comprised demographic information (e.g., age, body mass index [BMI]), obstetric history (e.g., parity, gestational week), clinical characteristics (e.g., cervical dilation, fetal heart rate baseline, PIH, gestational diabetes mellitus [GDM], C-reactive protein [CRP], neutrophil-to-lymphocyte ratio [NLR], estimated pelvic width, ultrasound cervical length, amniotic fluid index, body temperature, premature rupture of membranes [PROM], Bishop score), and psychological assessments (e.g., anxiety score, pain catastrophizing, visual analog scale [VAS] baseline). The outcome variable, efficacy of intrapartum labor analgesia, was defined as a binary outcome: 0 for effective analgesia (adequate pain relief without the need for rescue analgesia) and 1 for ineffective analgesia (breakthrough pain or requirement for rescue analgesia).

### Statistical analysis and model development

All statistical analyses were executed using R statistical software. Data preprocessing included the rigorous evaluation of missing and outlier values. Variables with a missing data rate exceeding 20% were excluded. For the remaining cohort, missing continuous data were addressed using median imputation, while categorical data were handled using mode imputation. Continuous variables were presented as medians with interquartile ranges (IQR) due to potential non-normal distribution, while categorical variables were reported as counts and percentages. Baseline characteristics are comprehensively summarized in Table 1.

**Table 1:** Baseline characteristics of the study cohort (*N* = 137).

Variable	Value (%)	*N*
Outcome		137
0	78 (56.93%)	
1	59 (43.07%)	
NLR	5.10 [3.70; 7.90]	137
VAS_Baseline	4.50 [1.90; 6.90]	137
Anxiety_Score	10.90 [5.20; 16.40]	137
Pain_Catastrophizing	26.70 [14.10; 39.90]	137
BMI	26.70 [23.60; 30.20]	137
Fetal_Estimated_Weight	3583.80 [3038.40; 4052.20]	137
Ultrasound_Cervical_Length	29.60 [22.20; 34.50]	137
Body_Temperature	37.30 [36.70; 37.90]	137
Gestational_Week	39.70 [38.40; 40.70]	137
Cervical_Dilation	4.70 [2.40; 7.20]	137
Bishop_Score	6.80 [3.40; 9.70]	137
Fetal_Heart_Rate_Baseline	144.50 [118.10; 166.20]	137
Estimated_Pelvic_Width	10.30 [8.70; 11.20]	137
Parity		137
0	68 (49.64%)	
1	69 (50.36%)	
PIH		137
0	117 (85.40%)	
1	20 (14.60%)	
PROM		137
0	125 (91.24%)	
1	12 (8.76%)	
CRP_Binary		137
0	66 (48.18%)	
1	71 (51.82%)	
LYM_percent_Binary		137
0	97 (70.80%)	
1	40 (29.20%)	
NLR_Binary		137
0	22 (16.06%)	
1	115 (83.94%)	
VAS_Baseline_Binary		137
0	62 (45.26%)	
1	75 (54.74%)	
GDM		137
0	103 (75.18%)	
1	34 (24.82%)	
Pain_Catastrophizing_Binary		137
0	37 (27.01%)	
1	100 (72.99%)	
Anxiety_Score_Binary		137
0	28 (20.44%)	
1	109 (79.56%)	
BMI_Binary		137
0	76 (55.47%)	
1	61 (44.53%)	
Gestational_Week_Binary		137
0	81 (59.12%)	
1	56 (40.88%)	
Body_Temperature_Binary		137
0	77 (56.20%)	
1	60 (43.80%)	
Amniotic_Fluid_Index	14.90 [10.70; 19.50]	137
Cervical_Dilation_Binary		137
0	91 (66.42%)	
1	46 (33.58%)	
Fetal_Estimated_Weight_Binary		137
0	83 (60.58%)	
1	54 (39.42%)	
LYM_percent	29.60 [18.90; 40.70]	137
CRP	10.30 [6.00; 15.20]	137
Fetal_Heart_Rate_Baseline_Binary		137
0	74 (54.01%)	
1	63 (45.99%)	
Amniotic_Fluid_Index_Binary		137
0	120 (87.59%)	
1	17 (12.41%)	
Estimated_Pelvic_Width_Binary		137
0	90 (65.69%)	
1	47 (34.31%)	
Ultrasound_Cervical_Length_Binary		137
0	87 (63.50%)	
1	50 (36.50%)	

#### Feature selection

**Figure 1:**
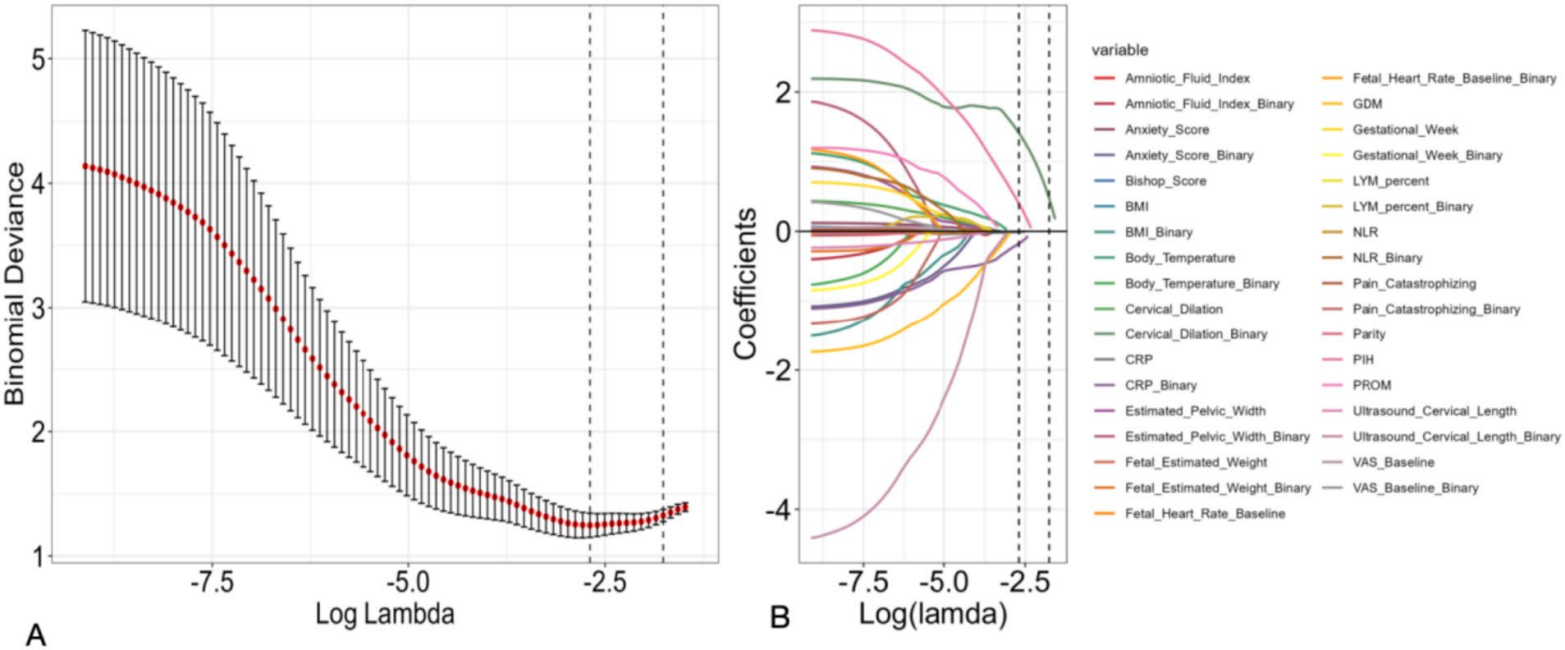
Feature selection using LASSO regression. **(A)** Ten-fold cross-validation plot of binomial deviance against log(*λ*), with the minimum deviance and 1-SE criteria indicated by dashed vertical lines. **(B)** LASSO coefficient profiles of the candidate predictors as a function of log(λ).

LASSO (Least Absolute Shrinkage and Selection Operator) regression was employed for feature selection to identify the most salient predictors and mitigate model complexity. The optimal lambda value was ascertained through cross-validation, as visually represented in Figure 1A. The evolution of coefficients with varying lambda values is depicted in Figure 1B illustrating the impact of regularization on feature importance.

#### Prediction model development

Five distinct machine learning algorithms were utilized for model development: Logistic Regression (LR), Naive Bayes (NB), Random Forest (RF), Support Vector Machine (SVM), and Extreme Gradient Boosting (XGB). The dataset was randomly partitioned into training and validation sets (e.g., 70% training, 30% validation) to ensure robust model assessment. To exclude potential heterogeneity bias, baseline characteristics between the training and validation sets were comprehensively compared. Continuous variables were compared using the Mann–Whitney U test, and categorical variables were assessed via the Chi-square test or Fisher’s exact test. The balance of baseline characteristics between the two sets is detailed in Supplementary Table S1.

### Model evaluation

**Figure 2:**
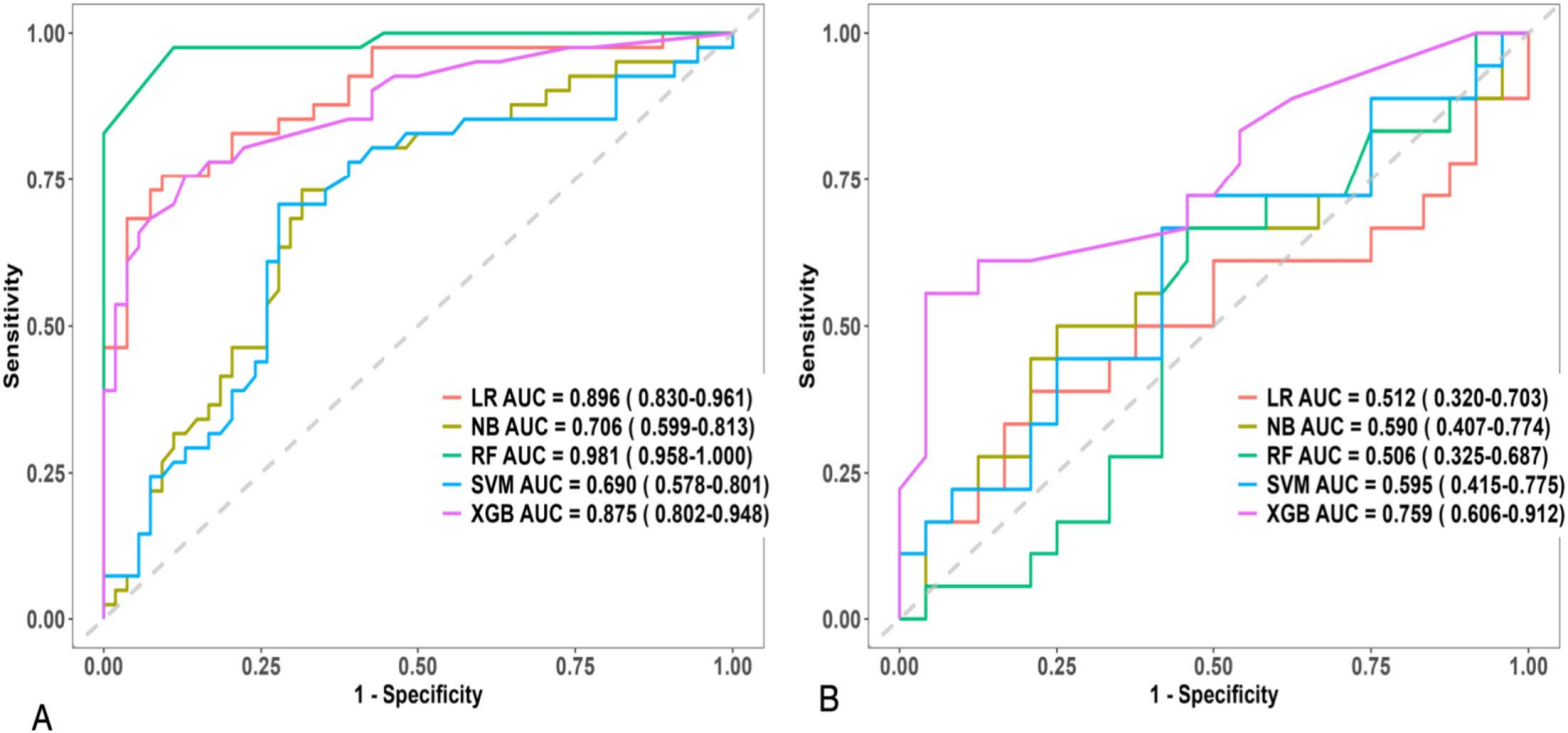
Receiver operating characteristic (ROC) curves of five machine learning models for predicting intrapartum labor analgesia efficacy. **(A)** Training set performance. **(B)** Validation set performance. AUC values with 95% confidence intervals are provided for Logistic Regression (LR), Naive Bayes (NB), Random Forest (RF), Support Vector Machine (SVM), and Extreme Gradient Boosting (XGB) models.

**Figure 3:**
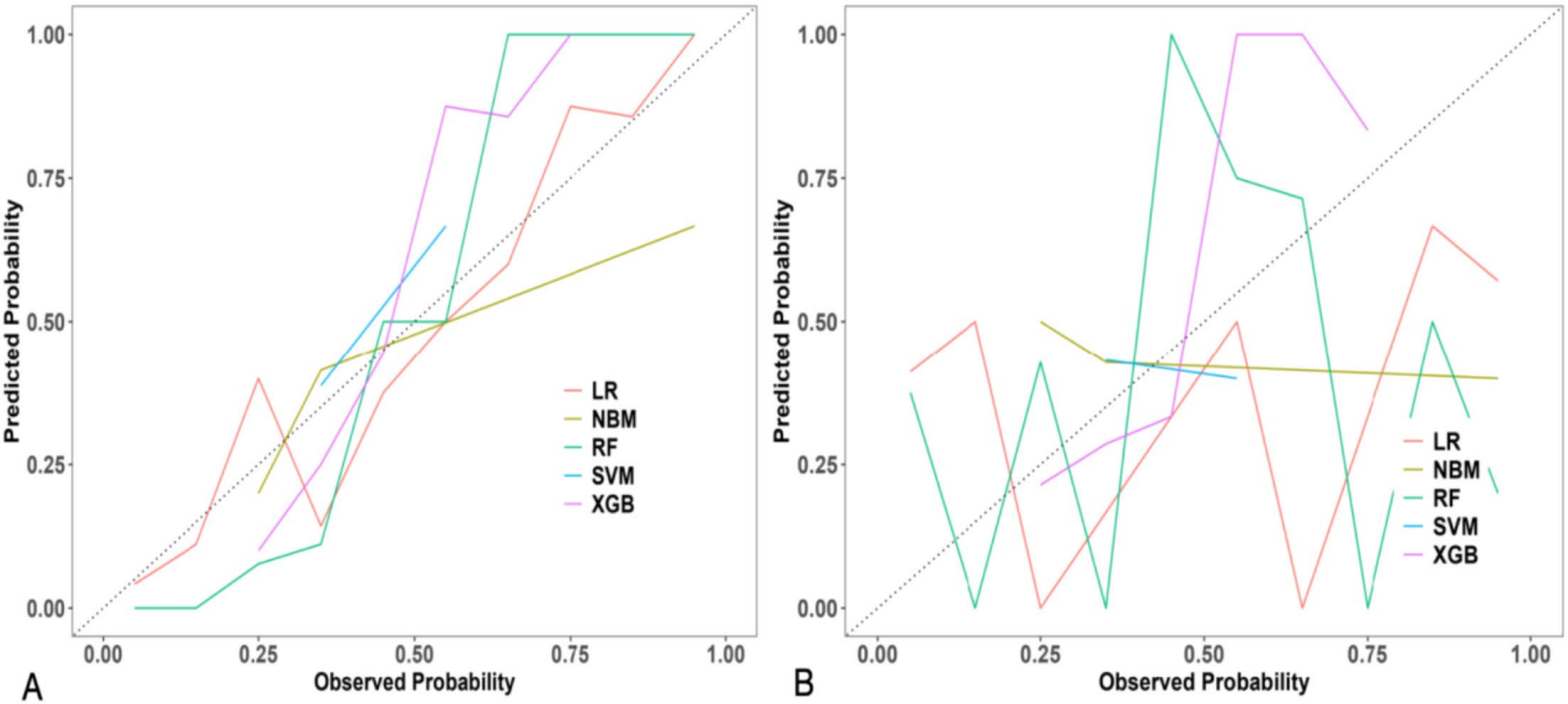
Calibration curves of five machine learning models for predicting intrapartum labor analgesia efficacy. **(A)** Training set. **(B)** Validation set. The diagonal dashed line represents perfect calibration, where predicted probabilities equal observed outcomes.

**Figure 4:**
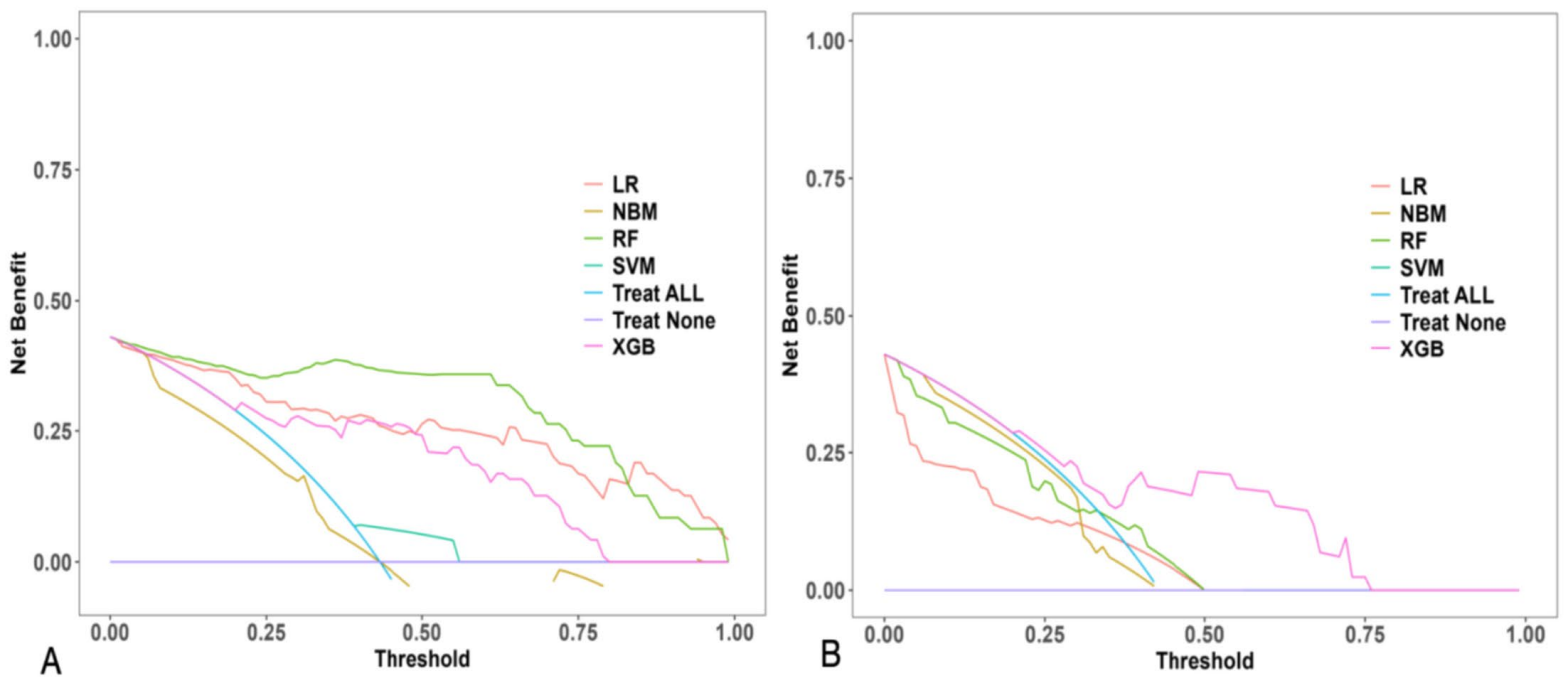
Decision curve analysis (DCA) of five machine learning models for predicting intrapartum labor analgesia efficacy. **(A)** Training set. **(B)** Validation set. Net benefit is plotted against a range of threshold probabilities. The “Treat All” and “Treat None” strategies are included for comparison.

**Figure 5:**
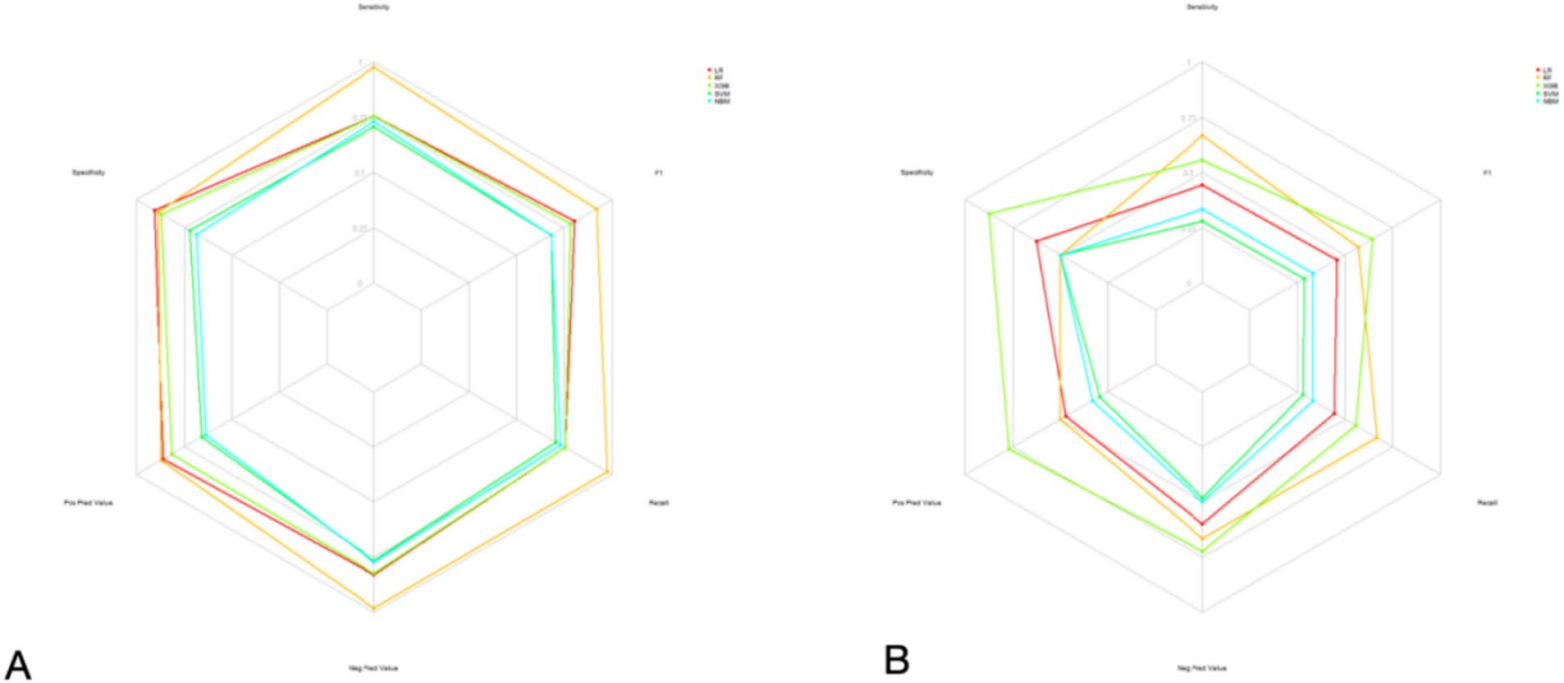
Radar plots of performance metrics for five machine learning models in predicting intrapartum labor analgesia efficacy. **(A)** Training set. **(B)** Validation set. Metrics include sensitivity, specificity, positive predictive value, negative predictive value, and F1 score.

**Figure 6:**
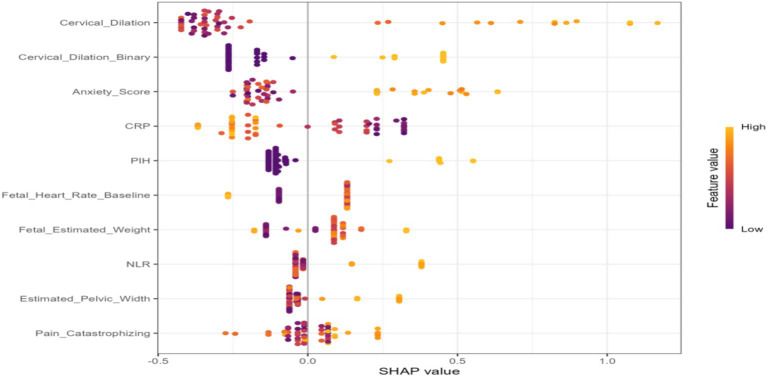
SHAP summary plot showing feature importance for the prediction of intrapartum labor analgesia efficacy. Each dot represents a single patient. The color indicates the feature value (red = high, blue = low), and the position on the x-axis shows the SHAP value, reflecting the contribution of the feature to the model prediction.

**Figure 7:**
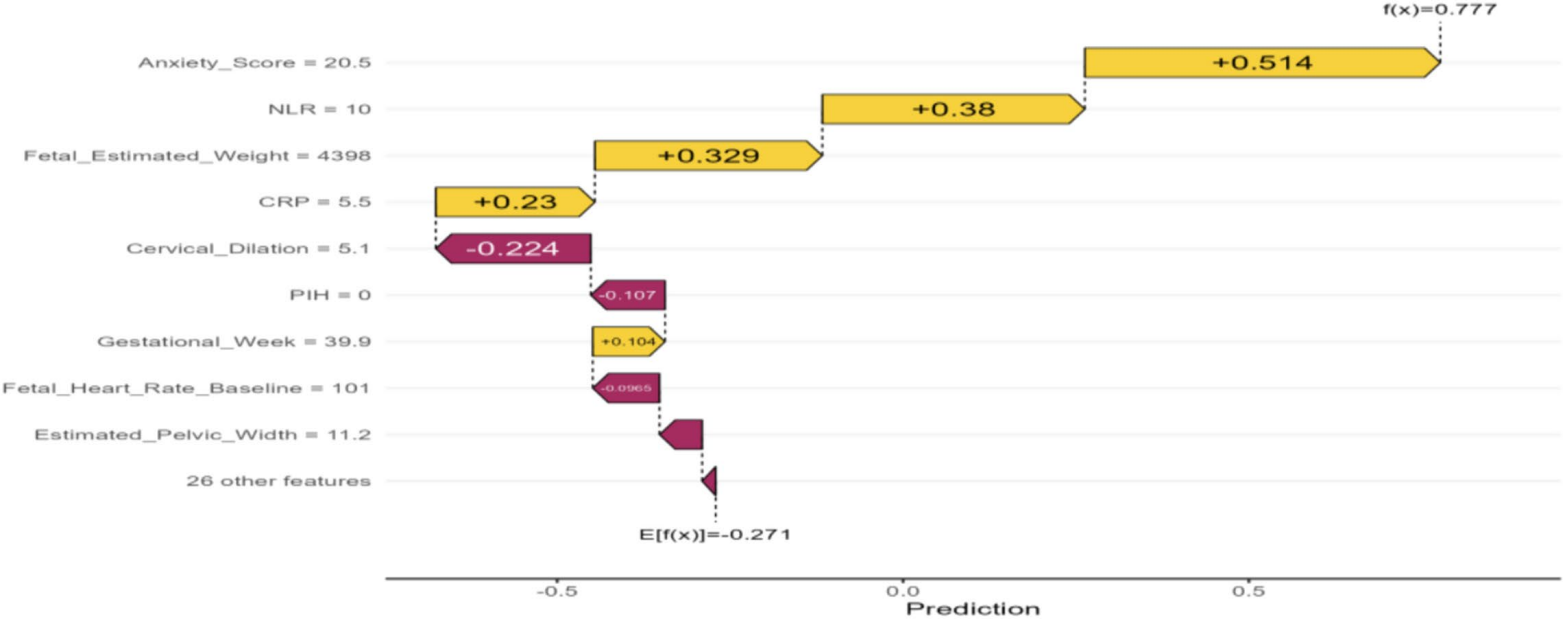
SHAP waterfall plot illustrating the contribution of individual features to the prediction of intrapartum labor analgesia efficacy for a representative case. Positive SHAP values (yellow) increase the predicted probability, while negative SHAP values (purple) decrease it.

Model performance was rigorously assessed using a suite of metrics:

- Discrimination: Receiver Operating Characteristic (ROC) curves and their corresponding Area Under the Curve (AUC) values were employed to evaluate the models’ capacity to differentiate between parturients experiencing effective versus ineffective analgesia (Figure 2).
- Calibration: Calibration curves were generated to ascertain the concordance between predicted probabilities and observed outcomes (Figure 3).
- Clinical Utility: Decision curve analysis (DCA) was conducted to appraise the clinical net benefit of employing the prediction models across a spectrum of threshold probabilities (Figure 4).
- Overall Performance: Sensitivity, specificity, positive predictive value (PPV), negative predictive value (NPV), and F1 score were also computed and visually presented using radar charts (Figure 5).
- Feature Importance and Interpretability: SHAP (SHapley Additive exPlanations) values were leveraged to interpret the contribution of individual features to model predictions, thereby enhancing model transparency (Figures 6, 7).

## Results

### Baseline characteristics

The baseline characteristics of the study cohort (*N* = 137) are summarized in Table 1. The outcome variable indicated that 56.93% (*n* = 78) of parturients achieved effective analgesia (outcome 0), while 43.07% (*n* = 59) experienced ineffective analgesia (outcome 1). Key continuous variables, such as NLR and VAS_Baseline, are presented with their medians and interquartile ranges.

### Feature selection

LASSO regression was applied for feature selection. Figure 1 graphically illustrates the binomial deviance as a function of log(lambda), pinpointing the optimal lambda value that minimizes deviance and elucidates the shrinkage of coefficients for various predictors as lambda increases, thereby highlighting the most influential features for the prediction model. LASSO regression refined the feature set, ultimately selecting 11 critical predictors for model construction. The complete list of features included in the final model comprises: PIH, ultrasound cervical length (binary and continuous), GDM, Anxiety Score, Neutrophil to Lymphocyte Ratio, C Reactive Protein, Cervical Dilation, Fetal Estimated Weight, Fetal Heart Rate Baseline, Estimated Pelvic Width, and Pain Catastrophizing. These were identified as important features, consistent with the initial logistic regression findings.

### Prediction model performance

Discriminative Ability: The Receiver Operating Characteristic (ROC) curves and their corresponding Area Under the Curve (AUC) values for the five machine learning models are presented in Figure 2. In Figure 2B, the XGB model exhibited the highest AUC of 0.759 (95% CI: 0.606–0.912), closely followed by SVM with an AUC of 0.595 (95% CI: 0.415–0.775).

#### Calibration

The calibration curves, depicted in Figure 3, illustrate the congruence between predicted probabilities and observed outcomes. While certain models demonstrated satisfactory calibration, others displayed deviations, indicating potential over- or under-prediction within specific probability ranges. For instance, Figure 3 suggests that the LR model generally overestimates probabilities at lower observed probabilities and underestimates at higher observed probabilities, whereas the XGB model exhibits superior calibration across a broader spectrum.

#### Clinical utility

Decision curve analysis (DCA) was executed to assess the clinical net benefit of the models. As evidenced in Figure 4, the XGB model consistently yielded a higher net benefit across a wide range of threshold probabilities when compared to other models and the treat-all or treat-none strategies. This finding implies that the application of the XGB model for prediction could lead to enhanced clinical decision-making.

#### Overall performance metrics

Radar charts in Figure 5 offer a comprehensive overview of model performance across multiple metrics, including sensitivity, specificity, F1 score, positive predictive value, and negative predictive value. The XGB model generally demonstrated a balanced and robust performance across these diverse metrics, further substantiating its efficacy.

### Feature importance and interpretability

SHAP values were employed to elucidate the contribution of individual features to the model’s predictions. Figure 6 (SHAP Summary Plot) furnishes a global perspective on feature importance, illustrating the distribution of SHAP values for each feature across all instances. This plot unequivocally confirms that features such as Anxiety_Score, NLR, CRP, and PIH are among the most influential predictors within the model. Figure 7 (SHAP Waterfall Plot) visually represents how each feature incrementally contributes to a single prediction, shifting it from the base value to the ultimate output. For instance, elevated Anxiety_Score and NLR values tend to augment the predicted probability of a specific outcome, whereas cervical dilation might exert a diminishing effect.

## Discussion

This study successfully developed and rigorously validated a machine learning-based prediction model for intrapartum labor analgesia efficacy, leveraging preoperative multidimensional indicators. Our findings unequivocally demonstrate that a judicious combination of demographic, clinical, and psychological factors can effectively forecast the success of labor analgesia. The XGB model consistently surpassed other traditional and advanced machine learning models across a spectrum of evaluation metrics, including AUC, calibration, and clinical net benefit, thereby strongly advocating for its potential clinical applicability.

The identification of pivotal predictors through LASSO regression and SHAP analysis provides profound insights into the intricate factors governing labor analgesia efficacy. Specifically, PIH, ultrasound cervical length, anxiety score, and NLR emerged as significant predictors. These observations resonate with existing literature, which posits that both physiological and psychological factors exert a substantial influence on pain perception and responsiveness to analgesia (10, 11). The capability to preoperatively identify parturients at an elevated risk of inadequate analgesia facilitates proactive interventions, such as tailored patient education, enhanced psychological support, or the consideration of alternative pain management modalities, ultimately leading to improved maternal satisfaction and safety (12, 13).

The superior performance of the XGB model can be ascribed to its inherent ability to adeptly manage complex non-linear relationships and intricate interactions among features, a common limitation encountered by conventional statistical models like logistic regression (14, 15). This finding aligns with recent predictive modeling studies in obstetric anesthesia, which also reported that ensemble learning methods, particularly XGBoost, frequently outperform traditional regressions in capturing multifaceted clinical data (16, 17). Furthermore, the integration of SHAP values significantly augments the interpretability of our model, offering clinicians a transparent understanding of how each patient’s unique profile contributes to their predicted outcome (10, 18, 19). This transparency is indispensable for fostering the adoption of AI-driven tools within clinical practice (3).

### Limitations

This study is not without limitations. Firstly, its retrospective design inherently introduces the potential for selection bias (5, 6). Secondly, the relatively modest sample size of 137 parturients may circumscribe the generalizability of our findings. However, given the exploratory nature of applying machine learning to multifaceted clinical data, we employed rigorous cross-validation and independent validation set testing to strictly mitigate the risk of overfitting, thereby ensuring the statistical reliability of our models within the current cohort. Thirdly, a standardized and universally accepted definition of labor analgesia efficacy remains an ongoing challenge across studies (11). Future research should prioritize prospective studies with larger and more diverse cohorts to rigorously validate our findings and further refine the prediction model (1, 2). Moreover, external validation in varied clinical settings would substantially fortify the evidence base for its clinical utility.

## Conclusion

We successfully developed a robust machine learning model, specifically an XGB-based model, that effectively predicts intrapartum labor analgesia efficacy using preoperative multidimensional indicators. This model represents a promising tool for personalized pain management during labor, empowering clinicians to identify high-risk parturients and implement precisely tailored interventions. Future research should concentrate on prospective validation and seamless integration of this model into routine clinical workflows to optimize maternal outcomes and satisfaction.

## Data Availability

The original contributions presented in the study are included in the article/Supplementary material, further inquiries can be directed to the corresponding author.

## Glossary

NLR: Neutrophil-to-Lymphocyte Ratio
CRP: C-Reactive Protein
LYM%: Lymphocyte Percentage
VAS: Visual Analog Scale (Baseline)
GAD-7: Generalized Anxiety Disorder-7 (Anxiety Score)
PCS: Pain Catastrophizing Scale
BMI: Body Mass Index
GW: Gestational Week
Parity: Parity (0 = Nulliparous, 1 = Multiparous)
BT: Body Temperature
CD: Cervical Dilation
FEW: Fetal Estimated Weight
UCL: Ultrasound Cervical Length
AFI: Amniotic Fluid Index
FHR: Fetal Heart Rate Baseline
EPW: Estimated Pelvic Width
GDM: Gestational Diabetes Mellitus
PIH: Pregnancy-Induced Hypertension
PROM: Premature Rupture of Membranes
BS: Bishop Score
Outcome: Analgesia Efficacy
