# Supplementary material for: Prediction model for intrapartum labor analgesia efficacy based on preoperative multidimensional indicators
Source: Front Med (Lausanne). 2026 May 19;13:1794753. doi: 10.3389/fmed.2026.1794753 (PMC13226191; doi:10.3389/fmed.2026.1794753)
Supplement: Supplementary file 1 [file Table_1.DOCX]

Table S1: Baseline Characteristics Comparison between Training and Validation Sets

| Variable | Training Set (N=95) | Validation Set (N=42) | *P*-value |
| --- | --- | --- | --- |
| AGE | 30.00 [24.00 - 35.00] | 28.00 [24.25 - 35.00] | 0.881 |
| NLR | 4.50 [2.25 - 7.30] | 5.05 [2.55 - 6.88] | 0.69 |
| NLR_Binary | 63 (66.3%) | 31 (73.8%) | 0.502 |
| CRP | 9.30 [6.25 - 14.50] | 9.30 [3.70 - 14.38] | 0.552 |
| CRP_Binary | 44 (46.3%) | 19 (45.2%) | 1 |
| LYM_percent | 28.90 [19.50 - 38.50] | 32.65 [20.02 - 38.68] | 0.651 |
| LYM_percent_Binary | 26 (27.4%) | 11 (26.2%) | 1 |
| Bishop_Score | 6.80 [3.65 - 10.60] | 7.15 [3.60 - 10.20] | 0.79 |
| VAS_Baseline | 5.30 [2.50 - 7.25] | 5.60 [2.40 - 7.20] | 0.834 |
| VAS_Baseline_Binary | 59 (62.1%) | 25 (59.5%) | 0.924 |
| Anxiety_Score | 10.40 [7.25 - 15.90] | 5.10 [2.02 - 10.07] | <0.001 |
| Anxiety_Score_Binary | 81 (85.3%) | 25 (59.5%) | 0.002 |
| Pain_Catastrophizing | 24.70 [10.45 - 37.00] | 23.85 [14.05 - 36.07] | 0.957 |
| Pain_Catastrophizing_Binary | 65 (68.4%) | 30 (71.4%) | 0.88 |
| BMI | 25.80 [21.75 - 29.85] | 26.05 [22.62 - 29.58] | 0.887 |
| BMI_Binary | 34 (35.8%) | 14 (33.3%) | 0.933 |
| Gestational_Week | 39.10 [37.95 - 40.40] | 39.10 [38.12 - 40.65] | 0.97 |
| Gestational_Week_Binary | 27 (28.4%) | 13 (31.0%) | 0.923 |
| Parity | 48 (50.5%) | 17 (40.5%) | 0.368 |
| Body_Temperature | 37.30 [36.60 - 37.85] | 37.20 [36.80 - 37.80] | 0.48 |
| Body_Temperature_Binary | 44 (46.3%) | 15 (35.7%) | 0.333 |
| Cervical_Dilation | 5.10 [2.65 - 7.70] | 5.95 [3.67 - 7.83] | 0.433 |
| Cervical_Dilation_Binary | 43 (45.3%) | 21 (50.0%) | 0.744 |
| Fetal_Estimated_Weight | 3519.50 [2967.90 - 3966.85] | 3586.30 [3056.10 - 3927.35] | 0.801 |
| Fetal_Estimated_Weight_Binary | 34 (35.8%) | 16 (38.1%) | 0.947 |
| Ultrasound_Cervical_Length | 26.70 [21.75 - 33.40] | 28.40 [20.97 - 32.58] | 0.582 |
| Ultrasound_Cervical_Length_Binary | 38 (40.0%) | 17 (40.5%) | 1 |
| Amniotic_Fluid_Index | 14.20 [10.45 - 19.10] | 16.15 [9.30 - 20.40] | 0.587 |
| Amniotic_Fluid_Index_Binary | 9 (9.5%) | 8 (19.0%) | 0.198 |
| Fetal_Heart_Rate_Baseline | 142.10 [120.10 - 155.15] | 140.50 [124.45 - 161.80] | 0.544 |
| Fetal_Heart_Rate_Baseline_Binary | 31 (32.6%) | 16 (38.1%) | 0.67 |
| Estimated_Pelvic_Width | 10.20 [9.50 - 11.20] | 10.15 [9.00 - 10.88] | 0.245 |
| Estimated_Pelvic_Width_Binary | 24 (25.3%) | 15 (35.7%) | 0.296 |
| GDM | 23 (24.2%) | 6 (14.3%) | 0.278 |
| PIH | 9 (9.5%) | 2 (4.8%) | 0.503 |
| PROM | 8 (8.4%) | 4 (9.5%) | 1 |
